# RASSF9 promotes NSCLC cell proliferation by activating the MEK/ERK axis

**DOI:** 10.1038/s41420-021-00583-0

**Published:** 2021-07-31

**Authors:** Jun Yuan, Qianqian Ju, Jun Zhu, Yun Jiang, Xuechao Yang, Xiaoyu Liu, Jinyu Ma, Cheng Sun, Jiahai Shi

**Affiliations:** 1 Nantong Key Laboratory of Translational Medicine in Cardiothoracic Diseases, Nantong Clinical Medical Research Center of Cardiothoracic Disease, and Institution of Translational Medicine in Cardiothoracic Diseases, Affiliated Hospital of Nantong University, Nantong, China; 2 Key Laboratory for Neuroregeneration of Jiangsu Province and Ministry of Education, Nantong University, Nantong, China; 3 NMPA Key Laboratory for Research and Evaluation of Tissue Engineering Technology Products, Nantong University, Nantong, China

**Keywords:** Cell growth, Non-small-cell lung cancer

## Abstract

The RAS-associated domain family 9 (RASSF9), a RAS-associated domain family gene, is expressed in a variety of tissues. However, its roles in tumorigenesis, particularly in non-small cell lung cancer (NSCLC), are still not understood well. In the present study, we aimed to examine the potential roles of RASSF9 in NSCLC and the underlying mechanisms. Our data showed that RASSF9 expression was upregulated in NSCLC tissues and cell lines. Increased expression of RASSF9 promotes NSCLC cell proliferation. On the contrary, knockdown of RASSF9 represses cell proliferation. Moreover, the effects of RASSF9 on NSCLC cell proliferation were further confirmed in vivo by using a subcutaneous tumor model. Mechanistically, pharmacological intervention studies revealed that the MEK/ERK axis is targeted by RASSF9 for transducing its regulatory roles on NSCLC cell proliferation. Collectively, our data indicate that RASSF9 plays a key role in tumorigenesis of NSCLC by stimulating tumor cell proliferation, which relies on activation of the MEK/ERK axis. Thus, RASSF9 might be a druggable target for developing novel agents for treating NSCLC.

## Introduction

Lung cancer is the most common cancer and a major cause of cancer-related death worldwide according to the newly report from the Global Cancer Statistics 2018 [1]. Non-small cell lung cancer (NSCLC) is the most common type, accounting for 85% of all lung cancer cases [2]. NSCLC can be further subdivided into three major pathologic subtypes, including lung adenocarcinoma, squamous cell carcinoma, and large cell carcinoma [3]. Lung adenocarcinoma is the most common subtype of NSCLC comprising ~40% of NSCLC cases, followed by squamous cell carcinoma 25–30% and large cell carcinoma 5–10% [4]. In recent decades, the clinical diagnosis and treatment of tumors have made great progresses due to the underlying molecular mechanisms for lung cancer development have been deeply studied. However, the long-term prognosis for NSCLC patients remains poor, with overall 5-year survival rates of <15% [5]. Hence, it is urgent to elucidate the detailed molecular pathogenesis of cell proliferation in NSCLC, so as to develop more effective therapeutic targets.

RASSF9 is a RAS-association domain family gene and is known to be expressed in multiple organs. The RASSF family includes 10 genes from RASSF1 to RASSF10 which contains a consensus RAS-association (RA) domain and bound to activated Ras [6, 7]. The RASSF family can be further subdivided into C-terminally (RASSF1-6) and N-terminally (RASSF7-10) based on whether there is a coiled-coil motif called the Salvador/RASSF/Hippo (SARAH) domain near the C-terminal or N-terminal RA domain [8, 9]. The N-terminal RASSF genes participate in numerous vital biological progresses, such as cell survival, apoptosis, tumor growth, and metastasis [7]. In addition, previous reports in the literature show RASSF7 expression is increased in NSCLC [10] and hepatocellular carcinoma [11] and plays a role in stimulating tumor proliferation. On the contrary, RASSF8 and RASSF10 have been shown to be reduced in multiple cancers, including lung cancer, gastric cancer, cervical cancer, breast cancer, colorectal esophageal squamous cell carcinoma, and hepatocarcinoma, and thus are considered as tumor suppressors [12–21]. However, the exact roles of RASSF9 in tumorigenesis in a variety of cancers, especially in NSCLC, are not fully understood.

In the present study, we observed RASSF9 expression is increased in NSCLC tissues and cell lines. Increased expression of RASSF9 promotes NSCLC cell proliferation, whereas knockdown of RASSF9 suppresses tumor cell proliferation. We further revealed that, by activating the MEK/ERK axis, RASSF9 plays a positive role in NSCLC cell proliferation. Our data suggest that RASSF9 might be a druggable target for developing novel agents for treating NSCLC.

## Results

### RASSF9 shows increased expression in NSCLC tissues and cell lines

**Fig. 1:**
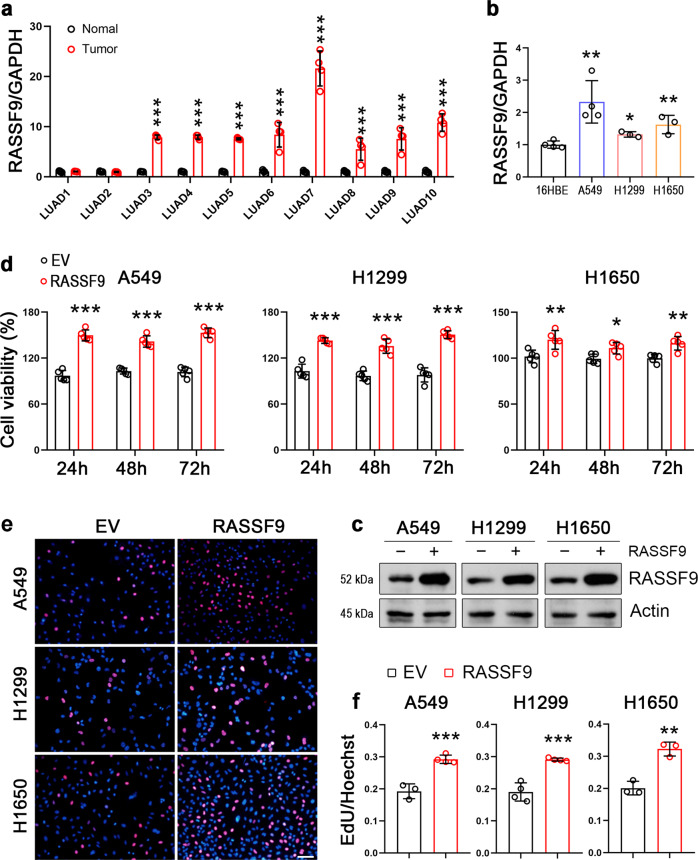
RASSF9 is highly expressed in NSCLC and plays a role in stimulating tumor cell proliferation. **a** The mRNA levels of RASSF9 in normal and NSCLC tissues. *n* = 10. **b** The mRNA levels of RASSF9 in human bronchial epithelial cells (16HBE) and NSCLC tumor cell lines (A549, H1299, and H1650). **c** RASSF9 was increased by the transfection with the plasmid carrying *Rassf9*. The control cells were transfected with the empty vector pcDNA3.1. Twenty-four hours post-transfection, cells were harvested for analyzing RASSF9 by western blot. Actin was used as a loading control. **d** RASSF9 improves cell viability. A549, H1299, and H1650 cells were transfected with plasmid expressing *Rassf9* and cell viability was analyzed by the method of MTT at different time points as indicated. The control cells were transfected with the empty vector pcDNA3.1 (EV). **e** RASSF9 promotes cell proliferation. A549, H1299, and H1650 cells were transfected with the plasmid expressing *Rassf9*. Twenty-four hours post-transfection, cell proliferation was analyzed by EdU incorporation assay. The control cells were transfected with the empty vector pcDNA3.1 (EV). Scale bar = 500 μm. **f** Quantitative analysis of EdU incorporation. Data are presented as means ± SD (error bars). *P < 0.05, **P < 0.01, and ***P < 0.001. Student’s *t*-test.

To investigate the potential role of RASSF9 in the development and progression of NSCLC, we first detected the mRNA levels of RASSF9 in clinical samples from NSCLC patients. Our results showed that, in eight paired tissues, RASSF9 was significantly increased in NSCLC tissues with the comparison of the adjacent normal tissues (Fig. 1a). In addition, we also measured RASSF9 expression in several non-small cell lung cancer cell lines, including A549, H1299, and H1650. As shown in Fig. 1b, we found that RASSF9 was markedly increased in A549, H1299, and H1650 as compared to 16HBE cells. Taken together, these data indicated that RASSF9 expression is increased in NSCLC, suggesting RASSF9 might play a crucial role in the progression of NSCLC.

### RASSF9 facilitates NSCLC cell viability and proliferation

**Fig. 2:**
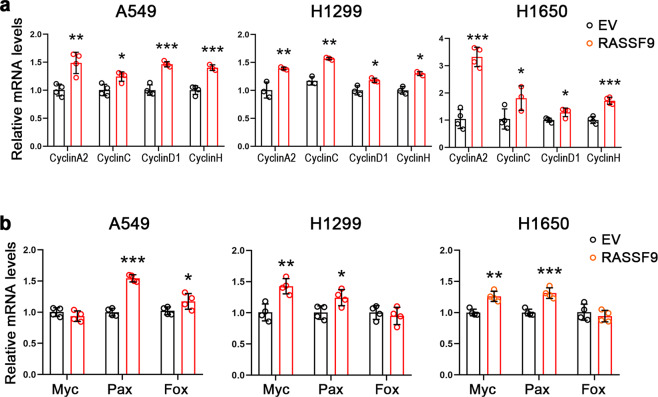
RASSF9 increases the expression of cyclins and oncogenes. **a** Elevated expression of RASSF9 increases the mRNA levels of cyclins in A549, H1299, and H1650 cells. **b** Effects of RASSF9 on the expression of *c-Myc*, *Pax*, and *Fos*. Cells were transfected with the plasmid expressing *Rassf9*. Control cells were transfected with the empty vector pcDNA3.1 (EV). Twenty-four hours post-transfection, total RNA was extracted for gene expression analysis by qRT-PCR, and GAPDH was used as a house-keeping gene. Data are presented as means ± SD (error bars). *P < 0.05, **P < 0.01, and ***P < 0.001. Student’s *t*-test.

We next examined the potential roles of RASSF9 on the progression of NSCLC such as cell proliferation. To this aim, cells were transfected with the plasmid carrying *Rassf9* to increase expression of RASSF9 (Fig. 1c). First, we observed that elevated RASSF9 expression caused appreciable growth in cell viability in these NSCLC cells (Fig. 1d). The EdU incorporation results showed that RASSF9 increased the cell proliferation in all three tested NSCLC cell lines including A549, H1299, and H1650 (Fig. 1e and f). Moreover, we also detected the expression of genes related to cell cycle and cell viability. The results showed that the mRNA levels of cyclins were markedly enhanced by RASSF9 in A549, H1299, and H1650 cells (Fig. 2a). We also detected the expression of three oncogenes (*c-Myc*, *Pax*, and *Fos*) and found that at least two genes were enhanced by RASSF9 in these NSCLC cells (Fig. 2b). These data indicate that RASSF9 has a positive regulatory role in cell proliferation in NSCLC.

### Knockdown of RASSF9 inhibits NSCLC cell viability and proliferation

**Fig. 3:**
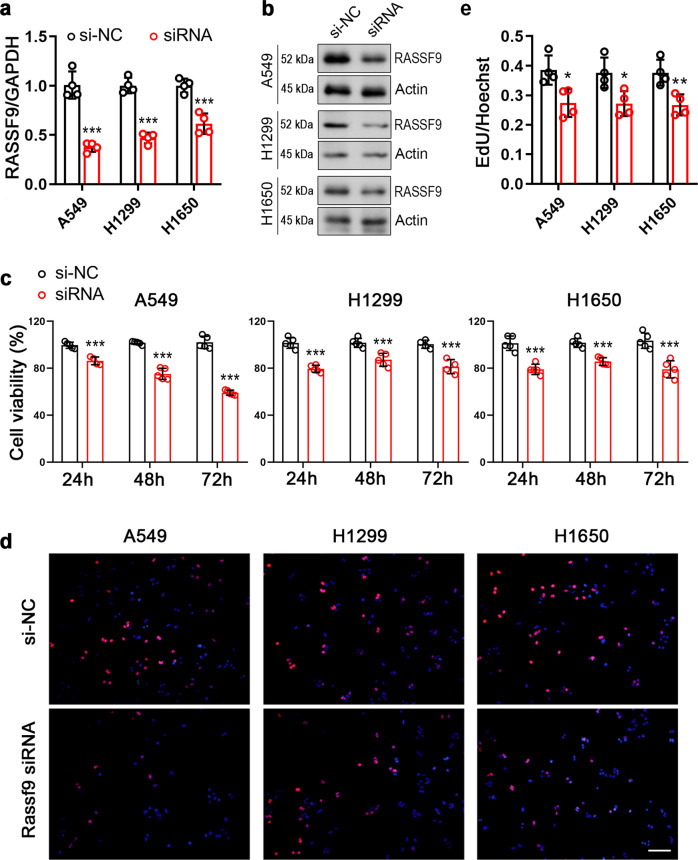
Knockdown of *Rassf9* suppresses NSCLC cell proliferation. **a** Knockdown of *Rassf9* by siRNA. A549, H1299, and H1650 cells were transfected with *Rassf9* siRNA or negative control (si-NC). Forty-eight hours post-transfection, total RNA was extracted for gene expression analysis by qRT-PCR and GAPDH was used as a house-keeping gene. **b** The protein levels of RASSF9 were reduced by siRNA. Cell treatments were described in (**a**). Protein levels were analyzed by western blot and Actin was used a loading control. **c** Knockdown of *Rassf9* decreases NSCLC cell viability. A549, H1299, and H1650 cells were transfected with *Rassf9* siRNA or si-NC. Cell viability was analyzed by the method of MTT at 24 h, 48 h, and 72 h post-transfection. **d** Knockdown of *Rassf9* reduces EdU incorporation. A549, H1299, and H1650 cells were transfected with Rassf9 siRNA or negative control (si-NC). Forty-eight hours post-transfection, cell proliferation was analyzed by EdU immunofluorescence assay. Scale bar = 500 μm. **e** Quantitative analysis of EdU incorporation. Data are presented as means ± SD (error bars). *P < 0.05, **P < 0.01, and ***P < 0.001. Student’s *t*-test.

**Fig. 4:**
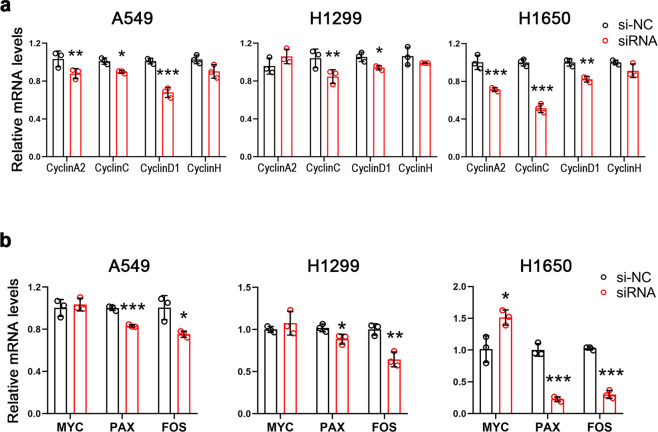
Knockdown of *Rassf9* decreases the expression of cyclins and oncogenes. **a** Knockdown of Rassf9 inhibits the mRNA levels of cyclins. **b** Effects of Rassf9 knockdown on the expression of *c-Myc*, *Pax*, and *Fos*. A549, H1299, and H1650 cells were transfected with *Rassf9* siRNA. Forty-eight hours post-transfection, total RNA was extracted for gene expression analysis by qRT-PCR and GAPDH was used as a house-keeping gene. Data are presented as means ± SD (error bars). *P < 0.05, **P < 0.01, and ***P < 0.001. Student’s *t*-test.

To further evaluate the significance of RASSF9 in the NSCLC progression, we downregulated RASSF9 expression in these NSCLC cells via small interference RNA (siRNA) (Fig. 3a, b). We found that siRNA-induced RASSF9 knockdown dramatically weakened cell viability (Fig. 3c). Furthermore, the results of EdU incorporation showed that decreased expression of RASSF9 markedly suppressed cell proliferation in A549, H1299, and H1650 cells (Fig. 3d, e). In addition, we also examined the mRNA levels of cyclins and the results showed that at least two cyclins were decreased by knockdown of RASSF9 in these NSCLC cells (Fig. 4a). Moreover, upon RASSF9 knockdown in these cells, the mRNA level of *Myc*, *Pax*, or *Fos* was significantly reduced (Fig. 4b). Collectively, these findings further indicate that RASSF9 plays a positive regulatory role on cell proliferation in NSCLC.

### RASSF9 stimulates cell proliferation through the MEK/ERK axis

**Fig. 5:**
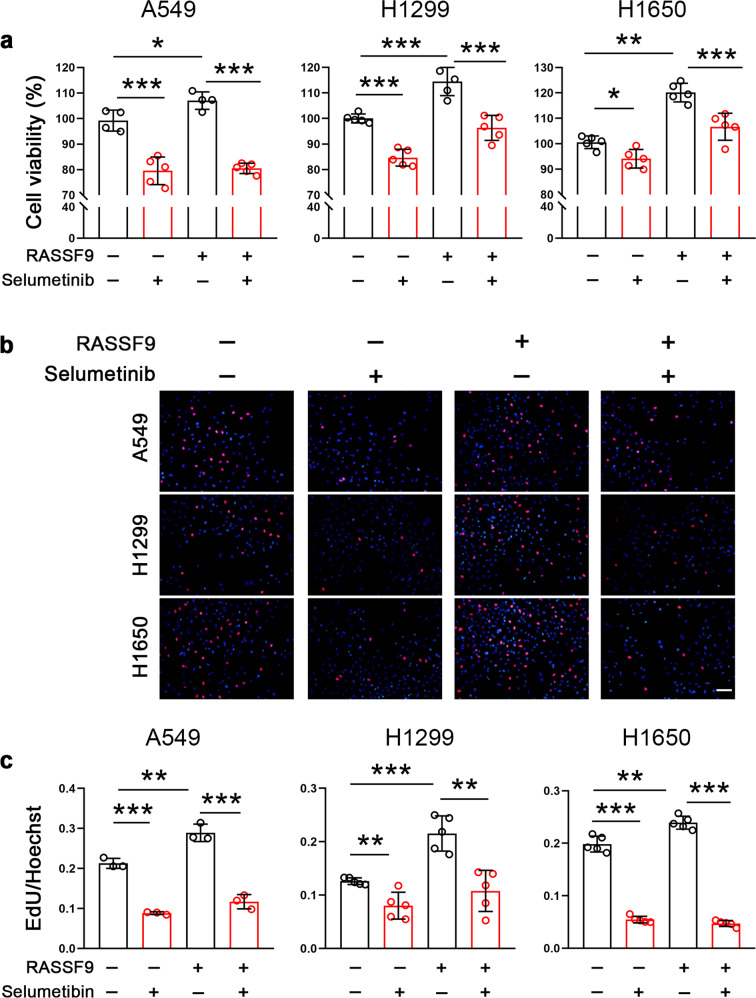
MEK inhibition by selumetinib reduces RASSF9-induced NSCLC cell viability and proliferation. **a** Selumetinib reduces RASSF9-induced upregulation of cell viability. A549, H1299, and H1650 cells were transfected with the plasmid expressing *Rassf9*. Twelve hours post-transfection, cells were treated with 10 μM of selumetinib for additional 24 h and then subjected for cell viability assay by the method of MTT. **b** Selumetinib decreases RASSF9-induced EdU incorporation. Cell treatments were described in (**a**). Scale bar = 500 μm. **c** Quantitative analysis for EdU incorporation. Data are presented as means ± SD (error bars). *P < 0.05, **P < 0.01, and ***P < 0.001. One-way ANOVA test.

**Fig. 6:**
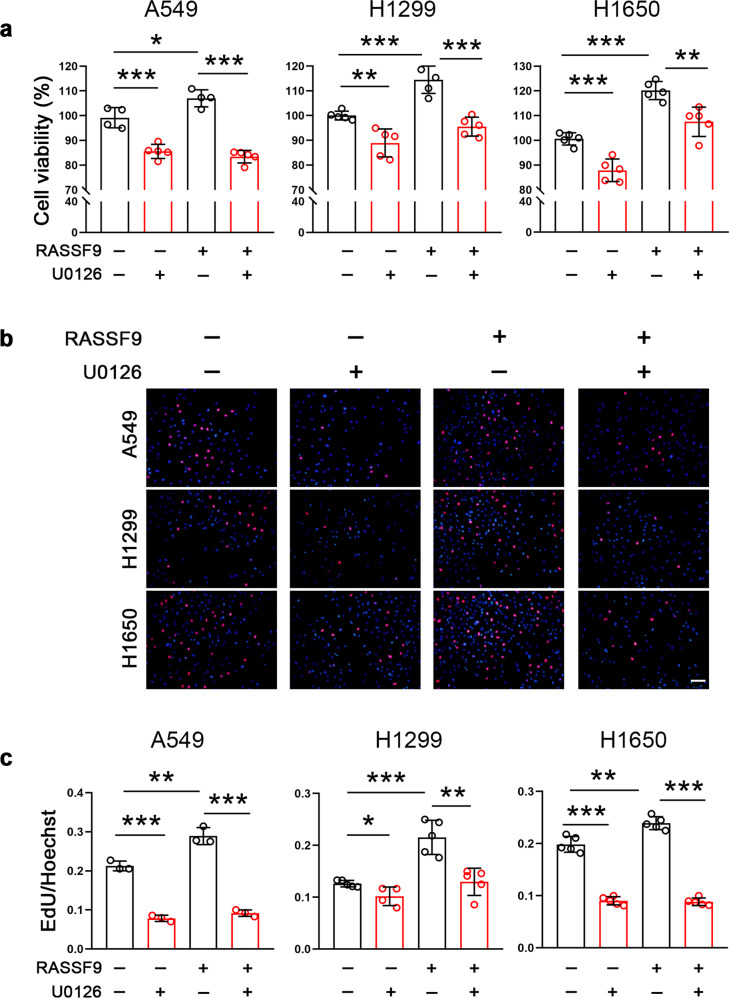
MEK inhibition by U0126 abolishes RASSF9-induced NSCLC cell viability and proliferation. **a** U0126 reduces the increased cell viability induced by RASSF9. A549, H1299, and H1650 cells were transfected with the plasmid expressing *Rassf9*. Twelve hours post-transfection, cells were treated with 10 μM of U0126 for additional 24 h and then subjected for cell viability assay by the method of MTT. **b** U0126 decreases RASSF9-induced EdU incorporation. Cell treatments were described in (**a**). Scale bar = 500 μm. **c** Quantitative analysis of EdU incorporation. Data are presented as means ± SD (error bars). *P < 0.05, **P < 0.01, and ***P < 0.001. One-way ANOVA test.

RASSF9 has been shown to positively regulate RAS signaling by inducing RAS dimerization, which activates downstream signal pathway including MEK and ERK to drive tumor cell proliferation [22]. Therefore, we next chose a pharmacological intervention strategy to examine the effects of RASSF9 on MEK/REK signal transduction. The results showed that selumetinib, a potent inhibitor of MEK, suppressed cell viability both in the presence or absence of RASSF9 in these NSCLC cells (Fig. 5a). Selumetinib also significantly reduced the EdU incorporation in A549, H1299, and H1650 cells (Fig. 5b, c). The increases in EdU incorporation induced by RASSF9 were also markedly blocked by selumetinib (Fig. 5b, c). Furthermore, we also chose another MEK inhibitor U1026 to confirm the above findings. As expected, the cell viability and the EdU incorporation were largely blocked by U0126 in A549, H1299, and H1650 cells (Fig. 6a–c).

**Fig. 7:**
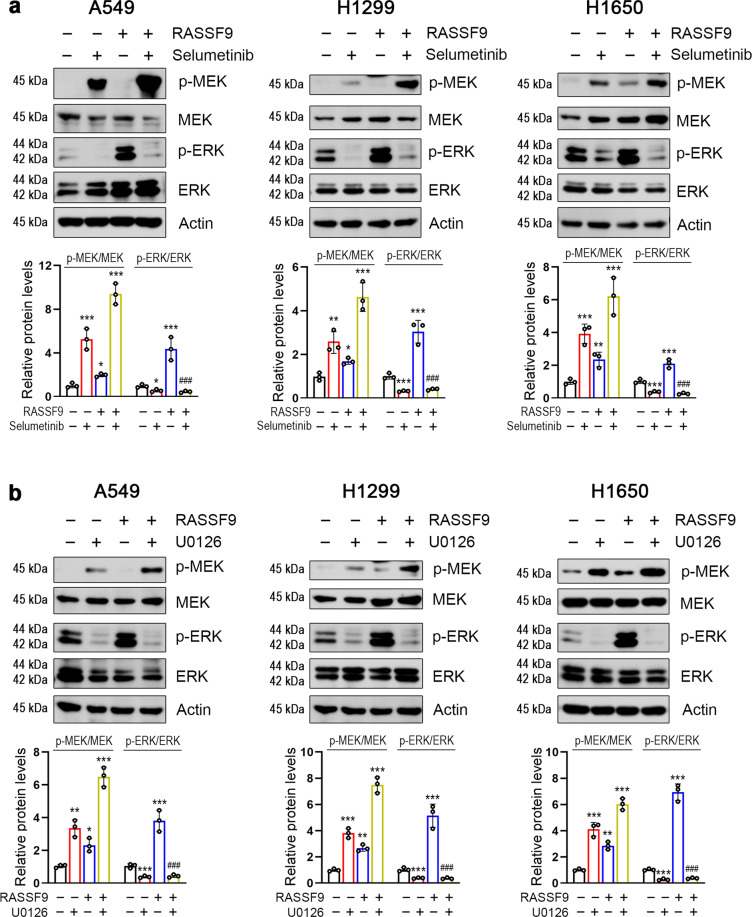
MEK inhibitors neutralize RASSF9-induced ERK activation. **a** Inhibition of MEK using selumetinib abolishes RASSF9-induced ERK activation. **b** Inhibition of MEK by U0126 reduces RASSF9-induced ERK activation. A549, H1299, and H1650 cells were transfected with the plasmid expressing *Rassf9*. Twelve hours post-transfection, cells were treated with 10 μM of U0126 or 10 μM of selumetinib for additional 24 h. Total cell lysates were prepared and subjected to western blot analysis. Actin was used as a loading control. Data are presented as means ± SD (error bars). *P < 0.05, **P < 0.01, and ***P < 0.001 versus the control cells. ^###^*P* < 0.001 versus the cells transfected with the plasmid expressing *Rassf9*. One-way ANOVA test.

Moreover, we also examined the protein levels involved in the axis of MEK/ERK to ascertain the inhibitory effects of selumetinib and U0126. As shown in Fig. 7a, the phospho-ERK (p-ERK) was robustly stimulated by RASSF9. In the presence of selumetinib, however, this stimulation was almost completely counteracted in all three tested NSCLC cells (Fig. 7a). The phospho-MEK (p-MEK) was also increased by RASSF9 (Fig. 7a). Selumetinib resulted in remarkable increases in p-MEK, similar findings were also reported previously [22, 23]. Similar to selumetinib, U0126 treatments also greatly blocked RASSF9 stimulated p-ERK in A549, H1299, and H1650 cells (Fig. 7b). Meanwhile, p-MEK was increased by U1026 (Fig. 7b). These data clearly indicated that the treatments of selumetinib and U0126 are effective strategies for blocking the axis of MEK/ERK induced by RASSF9 in NSCLC cells.

### RASSF9 stimulates NSCLC cell proliferation in vivo

**Fig. 8:**
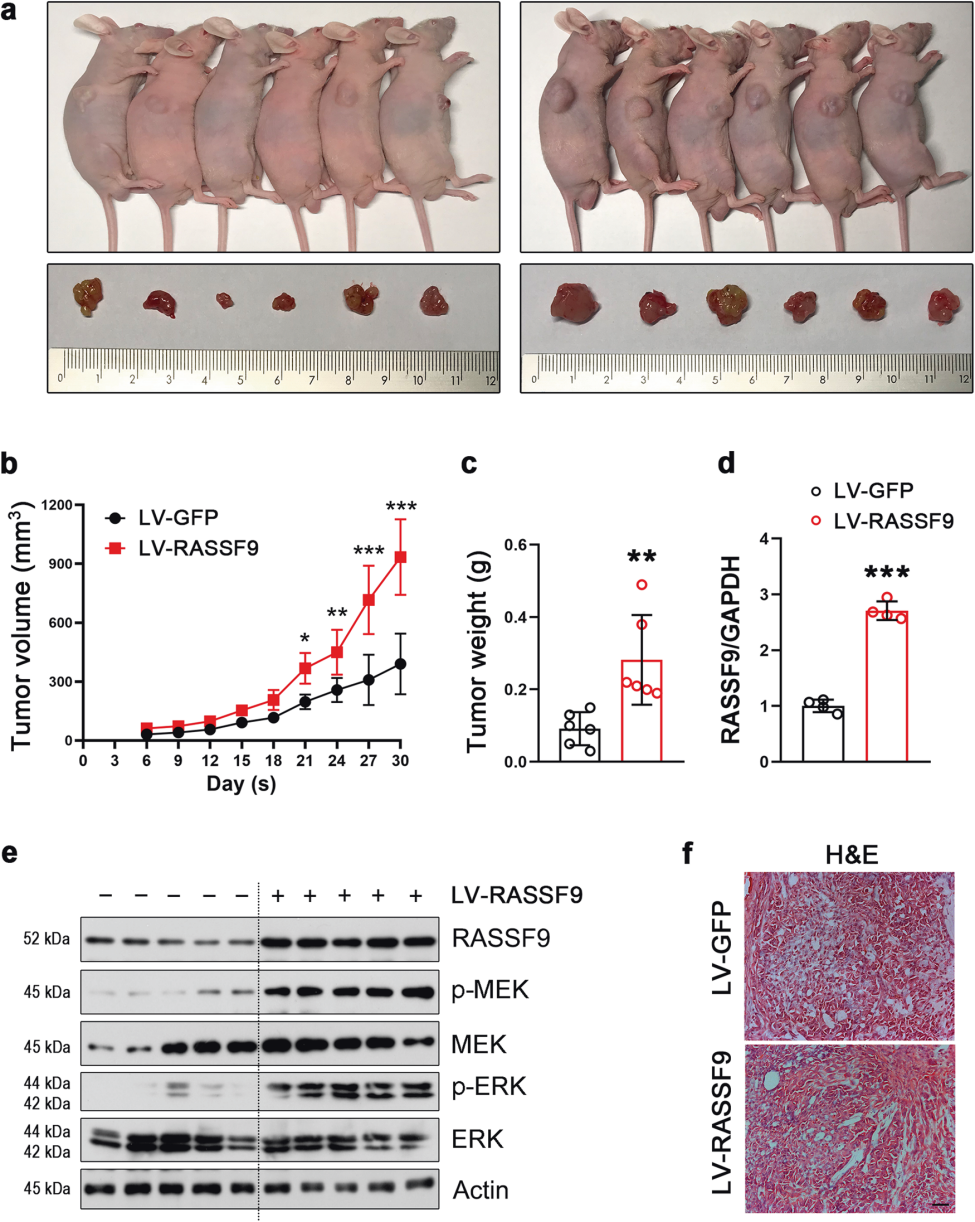
RASSF9 promotes NSCLC tumorigenesis in vivo. A549 cells were transduced with lentivirus expressing *Ras*sf9 (LV-RASSF9) or *Gfp* (LV-GFP). After screening with puromycin, the cells stably expressed RASSF9 or GFP were implanted into nude mice. **a** The representative images of gross subcutaneous tumors in nude mice and images of xenograft tumors dissected from nude mice (*n* = 6). **b** RASSF9 stimulates tumor volume (*n* = 6). **c** RASSF9 increases tumor weight (*n* = 6). **d** RASSF9 expression in the transplanted tumors was increased by LV-RASSF9 (*n* = 4). Gene expression was analyzed by qRT-PCR and GAPDH was used as house-keeping gene. **e** The protein levels of RASSF9, p-MEK, MEK, p-ERK, ERK in the transplanted tumors (*n* = 5). Protein levels were analyzed by western blot and Actin was used as loading control. **f** H&E staining. Scale bar = 200 μm. Data are presented as means ± SD (error bars). *P < 0.05, **P < 0.01, and ***P < 0.001. Student’s *t*-test.

We next attempted to determine whether RASSF9 positively regulates NSCLC cell proliferation in vivo. Therefore, we first established a stable cell line expressing RASSF9. To this aim, A549 cells were transduced with lentivirus carrying *Rassf9* (LV-RASSF9) and then subjected to puromycin screening. The resulted cells were injected subcutaneously into nude mice. Our results revealed that elevated expression of RASSF9 induced by LV-RASSF9 greatly stimulated cell growth, as evidenced by increased tumor mass, volume, and weight (Fig. 8a–c). Subsequently, RASSF9 expression in the LV-RASSF9 transduced tumors was confirmed by qRT-PCR (Fig. 8d). As expected, LV-RASSF9 induced an increase in RASSF9 protein levels, thereby stimulating an increase in p-MEK and p-ERK (Fig. 8e). Moreover, the hematoxylin/eosin (H&E) staining showed that tumor cells in the RASSF9 group were presented in nest-like distribution, and the nuclei were deeply stained, suggesting these cells grew vigorously (Fig. 8f). These in vivo data further confirmed the positive regulatory roles of RASSF9 on NSCLC cell proliferation.

## Discussion

RASSF9 is a member of RAS-association domain family. There is growing evidence that the dysregulation of RASSF9 expression is closely associated with many human diseases, including cancer [7, 24–26]. RASSF9 was initially identified as a protein based on interaction with the cytoplasmic domain of PAM (peptide acylglycine α-amino monooxygenase) through a yeast two-hybrid screen [27]. The follow-up study has shown that the protein encoded by this gene is located in the perinuclear endosomes [28]. This protein associates with PAM, and may be involved with the trafficking of this enzyme through secretory or endosomal pathways [28].

RASSF family is divided into two categories: C-terminal RASSFs (RASSF1-6) and N-terminal RASSFs (RASSF7-10) based on the location of the RA domain. Members of the C-terminal RASSF proteins are commonly considered as cancer suppressors and are involved in many important biological processes including regulation of apoptosis and proliferation, cell cycle, control of cell migration and adhesion, nuclear transport, and microtubule stability [29, 30]. RASSF7-10 is a newly identified family member that may also have important biological functions, some of which may be different from previously studied Ras effector molecules. Among them, RASSF7 regulates microtubule cytoskeleton and spindle formation, and plays a decisive role in mitosis and cell growth [31]. As a potential tumor suppressor gene, RASSF8 interacts with adherens junction component and adhesion-related β-catenin/E-cadherin to regulate cell adhesion function [12]. Moreover, lack of RASSF8 expression leads to increased cell migration and tumor aggression [32]. Likewise, RASSF10 also inhibits cell growth and colony formation, and induces apoptosis in various cell lines [33, 34]. RASSF9, a member of the N-terminal RASSF protein, has been shown to bind to Ras protein The RASSF9 and RAS family GTPases pull-down experiments indicate that RASSF9 binds to N-Ras, K-Ras, and R-Ras [34]. A previous study showed that RASSF9 induces dimerization of RAS, and eventually promotes cell growth in esophageal squamous cancer [22]. In line with this, our results suggest that RASSF9 expression is elevated in NSCLC tissues and cell lines. Meanwhile, the upregulation of RASSF9 expression enhanced the proliferation of tumor cells, while downregulation of RASSF9 resulted in the opposite outcome. In contrast, two recent studies have described that RASSF9 is decreased in breast and gastric cancer, and that RASSF9 suppresses the proliferation of these cancer cells [35, 36]. We speculated that the discrepancy is most likely due to different tumor cell types.

The RAS/RAF/MEK/ERK signaling cascade is a mitogen-activated protein kinase (MAPK) pathway that can be widely activated. It can transmit extracellular signals into the nucleus and cause changes in the expression profile of specific proteins in the cell, thereby affecting cell proliferation, differentiation, survival, and apoptosis [37, 38]. Once this pathway is activated, RAS combines with RAF family kinases to phosphorylate and activate RAF, MEK, and ERK kinases in turn. In addition, the latest evidence from current reports revealed elevated expression of RASSF9 lead to RAS dimer formation and RAF, MEK, and ERK activation [22]. In line with this view, in our study, we detected the expression of MEK, p-MEK, ERK, and p-ERK, and found that RASSF9 stimulated the p-MEK and p-ERK. Meanwhile, inhibition of MEK by selumetinib or U0126 eliminated the effects of RASSF9 on cell proliferation. These results suggest that RASSF9 may stimulate the proliferation of NSCLC cells by activating the MEK/ERK signaling pathway.

Taken together, our study revealed that expression of RASSF9 is upregulated in NSCLC tissues and cell lines. RASSF9 promotes the proliferation of NSCLC cells, while knockdown of RASSF9 inhibits the growth of tumor cells. In addition, we further confirm the roles of RASSF9 on the proliferation of NSCLC cells by using xenograft model. Pharmacological interventions revealed that activation of the MEK/ERK axis is responsible for RASSF9-induced tumor cell proliferation. Moreover, RASSF9 might be a druggable target for developing agents for treating NSCLC.

## Materials and methods

### Human specimens

To examine *Rassf9* expression, a cohort of 10 patients with lung adenocarcinoma patients were enrolled from the Affiliated Hospital of Nantong University (Nantong, China). The patients are varied from 60 to 75 years of age, male, and Chinese Han nationality. All diagnoses were pathologically confirmed. None of the patients had received any anti-tumor treatments such as radiotherapy, chemotherapy, and immunotherapy prior to the surgery. Following surgical excision, all the fresh tissues including lung adenocarcinoma and matched adjacent tissues were immediately frozen in liquid nitrogen and placed in −80 °C freezer for long-term storage. All patients received written informed consent. This study was approved by the Ethics Committee of Affiliated Hospital of Nantong University.

### Cell culture

All human NSCLC cell lines including A549, H1299, and H1650 were obtained from the National Collection of Authenticated Cell Cultures (Shanghai, China). 16HBE cells were from Sigma-Aldrich (City of Saint Louis, USA). Cells were cultured in DMEM supplemented with 10% FBS, 1% penicillin/streptomycin, and incubated at 37 °C in a humidified atmosphere with 5% CO_2_.

### Cell treatments

Cell transfection was achieved by using Lipofectamine RNAiMAX (Invitrogen) for siRNAs, or Lipofectamine 2000 (Invitrogen) for plasmids according to the manufacturer’s protocols. The *Rassf9* siRNA sequence and the plasmid expressing *Rassf9* were described previously [22]. Twenty-four hours post-transfection with plasmids or 48 h post-transfection with siRNAs, cells were then subjected to various experiments such as MTT, cell colony formation, or EdU incorporation assays. For pharmacological interventions, cells were treated with U0126 (10 μM) or selumetinib (10 μM) for additional 24 h.

### MTT assay

NSCLC cells (5000 cells/well in 100 µl DMEM) were seeded into 96-well plates and cultured for 24 h, followed by transfection with the plasmid expressing *Rassf9* or *Rassf9* siRNA. Cell viability was then examined by MTT assay with the manufacturer’s instructions. Cell growth was evaluated by the absorbance at 570 nM in a Microplate Reader (BioTek, Winooski, VT).

### EdU assay

Ten micromolar of EdU (5-ethynyl-2′-deoxyuridine) was added to cells and then cultured for 2 h at 37 °C. After fixation, cells were incubated in the Click reaction mixture, in which Alexa Fluor 594 was contained, for 30 min at room temperature in the dark, and then cells were washed with PBS thrice and stained with Hoechst (diluted 1:1000 with PBS) at room temperature for 10 min to label the nucleus. Finally, EdU incorporation was observed with a fluorescent microscope (Zeiss, Germany).

### Lentivirus transduction

The production of lentivirus expressing *Rassf9* (LV-RASSF9) was described previously [22]. Briefly, the coding sequences of *Rassf9* were synthesized and incorporated into pHBLV-CMV-MCS-3FLAG-EF1-ZsGreen-T2A-PURO at the sites of EcoR I and BamH I. The prepared plasmid was then transfected into 293T cells for producing lentivirus expressing *Rassf9* (LV-RASSF9). The plasmid construction and lentivirus generation were performed at HANBIO (Shanghai, China). A549 cells were transduced with LV-RASSF9 to overexpress RASSF9 (MOI = 10). The control cells were transduced with LV-GFP at the same dosage. Meanwhile, 5 µg/ml polybrene was added to improve the transduction efficiency. Transfected A549 cells were selected with 2.5 μg/ml puromycin for 1 week. The efficiency for lentivirus-mediated *Rassf9* expression in A549 was verified by qRT-PCR and western blot analysis. The cells stably expressed RASSF9 or GFP were used for tumor cell transplantation experiments.

### Animal experiments

Six-week-old male BALB/c nude mice (18–22 g) were purchased from Shanghai Slake Laboratory Animal Co. Ltd (Shanghai, China). Mice were bred and maintained in a specific pathogen-free (SPF) environment (group housing up to 5 mice per cage). Mice were randomly divided into two groups: LV-GFP group (*n* = 6) and LV-RASSF9 group (*n* = 6). No statistical methods were used to pre-determine sample size. The tumor model was established by hypodermic injection of 2 × 10^6^ A549 cells under the right axilla of BALB/C nude mice. Tumor growth was monitored every 3 days and measured using a vernier calipers. The volume of tumor based on caliper measurements was estimated by the formula: tumor volume = 1/2 length × width × width. After 1 month, all mice were sacrificed and tumor tissues were excised for further analysis. All experiments involving animals were approved by the Institutional Animal Care and Use Committees of the Nantong University (Approval ID: SYXK [SU] 2017-0046).

### Quantitative real time-PCR (qRT-PCR)

Total RNA was extracted from the lung cancer cells using TRIzol Reagent (Invitrogen) according to the manufacturer’s protocol. Then RNA quantity and quality were determined with a spectrophotometer (ND-1000; NanoDrop Technologies). cDNA was generated using a Prime Script RT Master Mix (Bio-Rad). The primer sequences are *Rassf9*, 5′-GAG GAC CTG AGC GAA AGT GAT-3′ (forward) and 5′-TCT GGA TGC CAC TCA AAT GAG A-3′ (reverse); *Fos*, 5′-GAT ACA CTC CAA GCG GAG AC-3′ (forward) and 5′-CCC AGT CAG ATC AAG GGA AG-3′ (reverse); *Myc*, 5′-CGA CTC TGA GGA GGA ACA AG-3′ (forward) and 5′-CGT AGT TGT GCT GAT GTG TG-3′ (reverse); *Pax*, 5′-ACC AAT CAG CAT AGG AAT CTG-3′ (forward) and 5′-TGC TGT TGT TGC TTG AAG AC-3′ (reverse); *Ccna2* (Cyclin A2), 5′-CGC TGG CGG TAC TGA AGT C-3′ (forward) and 5′-GAG GAA CGG TGA CAT GCT CAT-3′ (reverse); *Ccnc* (Cyclin C), 5′-CCT TGC ATG GAG GAT AGT GAA TG-3′ (forward) and 5′-AAG GAG GAT ACA GTA GGC AAA GA-3′ (reverse); *Ccnd1* (Cyclin D1), 5′-GCT GCG AAG TGG AAA CCA TC-3′ (forward) and 5′-CCT CCT TCT GCA CAC ATT TGA A-3′ (reverse); *Ccnh* (Cyclin H), 5′-TGT TCG GTG TTT AAG CCA GCA-3′ (forward) and 5′-TCC TGG GGT GAT ATT CCA TTA CT-3′ (reverse). *Gapdh*, 5′-GAC CTG ACC TGC CGT CTA-3′ (forward) and 5′-AGG AGT GGG TGT CGC TGT-3′ (reverse). qRT-PCR was performed using SYBR Premix Ex Taq II on a Lightcycler 96 system (Roche). Data were analyzed using the 2^−△△CT^ method.

### Protein extraction and western blot analysis

Cells or tissues were lysed with ice-cold lysis buffer (25 mM Tris-HCl, pH 7.4; 100 mM NaF; 50 mM Na_4_P_2_O_7_; 10 mM Na_3_VO_4_; 10 mM EGTA; 10 mM EDTA; 1% NP-40; 10 μg/ml Leupeptin; 10 μg/ml Aprotinin; 2 mM PMSF and 20 nM Okadaic acid). Protein concentrations were determined by BCA assay (Thermo Scientific) according to the manufacturer’s directions. The membranes were blocked for 1 h at room temperature in TBS-T (Tris-buffered saline + 0.05% Tween-20) containing 5% BSA and incubated overnight at 4 °C with the following primary antibody: rabbit anti-RASSF9 (Invitrogen; PA5-58785), anti-MEK1/2 (Cell Signal Technology; #9126), anti-phospho-MEK1/2 (Cell Signal Technology; #9154), anti-ERK1/2 (Cell Signal Technology; #9102), anti-phospho-ERK1/2 (Cell Signal Technology; #4370), and mouse anti-β-actin (Cell Signal Technology; #3700) was used as an internal control. Then followed by incubation with the secondary anti-rabbit (Santa Cruz Biotechnology; sc-2357) or anti-mouse (Santa Cruz Biotechnology; sc-2005) HRP-conjugated antibodies at room temperature for 1 h. Finally, protein signals were visualized using an enhanced chemilluminescense detection reagent (Roche). The western blot data were quantified by using the ImageJ software.

### H&E staining

H&E staining was performed with the following standard procedures. Briefly, the collected tumors from nude mice were fixed with 4% paraformaldehyde and dehydrated with successive sucrose gradient for 24–48 h. Then, the tissues were cut into 10-μm-thick frozen sections and processed for H&E staining. After washes, the slides were counterstained with haematoxylin for 5 min, rinsed with a continuous flow of running water and then immerged in hydrochloric-alcohol solution for 15 s. Thereafter, the slides were stained with 0.5% eosin for 30 s. Finally, the slides were dehydrated with ethanol, transparentized with xylene, and eventually sealed with neutral gum. After staining, the slides were analyzed and photographed under a Zeiss microscope.

### Statistical analysis

The investigators were not blind to the group allocation during the experiments. However, data collection and analysis were performed blindly. All results are expressed as the mean ± standard deviation (SD) of three independent experiments. Statistical analysis was conducted by GraphPad Prism version 8.0 (GraphPad Software, CA). Similar variances between the groups are being statistically compared. The data were analyzed by one-way ANOVA test or Student’s *t*-test. A value of *P* < 0.05 was accepted as statistical significance. No data were excluded in the analyses. No statistical methods were used to pre-determine sample size but our sample sizes are similar to those reported in our previous publications [39, 40].
